# Hepatitis B Virus Genotype D Isolates Circulating in Chapecó, Southern Brazil, Originate from Italy

**DOI:** 10.1371/journal.pone.0135816

**Published:** 2015-08-14

**Authors:** Carolina Souza Gusatti, Cintia Costi, Maria Laura Halon, Tarciana Grandi, Arlete Ferrari Rech Medeiros, Cláudia Maria Dornelles Silva, Selma Andrade Gomes, Marcia Susana Nunes Silva, Christian Niel, Maria Lucia Rosa Rossetti

**Affiliations:** 1 Programa de Pós-Graduação em Biologia Celular e Molecular, Centro de Biotecnologia, Universidade Federal do Rio Grande do Sul, Porto Alegre, Brazil; 2 Centro de Desenvolvimento Científico e Tecnológico, Fundação Estadual de Produção e Pesquisa em Saúde, Porto Alegre, Brazil; 3 Setor de Hepatites Virais, Secretaria Municipal de Saúde, Chapecó, Brazil; 4 Laboratório de Virologia Molecular, Fundação Oswaldo Cruz, Fiocruz, Rio de Janeiro, Brazil; 5 Programa de Pós-Graduação em Biologia Celular e Molecular Aplicada à Saúde, Universidade Luterana do Brasil, Canoas, Brazil; CRCL-INSERM, FRANCE

## Abstract

Hepatitis B virus genotype A1 (HBV/A1), of African origin, is the most prevalent genotype in Brazil, while HBV/F predominates in the other South American countries. However, HBV/D is the most common in the three states of southern Brazil, where ‘islands’ of elevated prevalence, as Chapecó and other cities, have been described. In this study, 202 HBV chronic carriers attending in 2013 the viral hepatitis ambulatory of Chapecó, were investigated. In comparison with previous studies performed in the same ambulatory, a rapid aging of the HBV infected population was observed (mean age of the newly diagnosed patients increasing from 29.9 ± 10.3 years in 1996 to 44.4 ± 13.3 years in 2013), probably due to a singular vaccination schedule at Chapecó that included not only children but also adolescents. Phylogenetic and BLAST analyses (S region) classified 91 HBV isolates into genotypes A (n = 3) and D (n = 88). The majority of HBV/D isolates were closely related to D3 sequences. To understand the reasons for the absence or near absence of genotypes A and F, and how HBV/D was introduced in the south of Brazil, HBV/D infected patients were inquired about their genealogical and geographical origins. Forty-three (52%) patients have their four grandparents of Italian origin, vs. seven (8%) who have their four grandparents of Brazilian origin. At all, 65 out of 83 (78%) patients had at least one grandparent originating from Italy. Taking into consideration the fact that Italy is one of the few countries where subgenotype D3 is predominant, the results strongly suggested that HBV/D was introduced in Brazil through Italian immigration which culminated between 1870 and 1920.

## Introduction

Despite the availability of a prophylactic vaccine for more than 20 years, hepatitis B remains one of the major public health problems worldwide. More than 240 million people are chronically infected with hepatitis B virus (HBV) and more than 780,000 die every year due to the acute or chronic consequences of hepatitis B [1]. HBV prevalence, measured by the presence of hepatitis B surface antigen (HBsAg) in the serum, varies from < 1% to > 10% depending on the country.

In Brazil, endemicity is low, and public immunization programs have been implemented since the early 1990s. The estimated HBsAg prevalence among people aged 20 to 69 years varies from 0.40% to 0.92% in the capitals of the 26 states [2]. However, some areas show prevalence rates markedly higher than the average, not only in the Amazon region, but also in some southern counties, as Chapecó [3,4] and Caixas do Sul [5].

HBV isolates have been classified into eight genotypes (A to H), based on a genomic sequence divergence > 7.5% over the entire DNA genome [6]. Additionally, genotypes I [7] and J [8] have been proposed. The most cosmopolitan genotypes are A and D. Genotypes B and C are found in East and Southeast Asia, genotype E in West Africa, and genotype F is spread among indigenous Americans [9]. Within genotype D, at least seven subgenotypes have been described. HBV/D1 is the most prevalent subgenotype in Greece, Turkey and North Africa, D2 in northeastern Europe (Russia, Belarus, Estonia) and Albania, and D3 in Italy and Serbia. The other subgenotypes circulate mainly outside Europe and the Americas [10,11]. Chronic patients infected with HBV/A show a favorable response to alfa-interferon more frequently than those infected with HBV/D (reviewed in [12]).

In South America, genotype A is predominant in Brazil [13], which is the only Portuguese speaking country, while genotype F has been shown to be the most prevalent in the other, Spanish speaking countries [14]. However, genotype D is widespread in southern Brazil. Recent studies have suggested that (i) the majority of HBV/A isolates circulating in Brazil (subgenotype A1) originated from the slaves removed from Southeast Africa at the middle of the 19th century [15], and (ii) HBV genotypes from European origin explained the elevated endemicity found in some southern Brazil areas [16].

In this study, 91 HBV isolates from chronic patients living in Chapecó (southern Brazil) were genotyped. Eighty-eight (97%) belonged to genotype D. In an attempt to understand the reasons of the absence or near absence of genotypes A and F in this population, and how HBV/D was introduced in the south of Brazil, the question of the genealogical and geographical origins of the patients was addressed, and a phylogenetic analysis was performed.

## Materials and Methods

### Ethics Statement

All subjects, who knew to be or have been infected with HBV, gave their written consent to participate to the study, and answered questions about age, ethnicity, health status, occupation, lifestyle including drug use and sexual behaviour, blood transfusion, surgery, hemodialysis, presence of HBV carriers in the family, and others allowing to evaluate risk factors for HBV infection. This study was approved by the Ethics in Research Committee of Fundação Estadual de Produção e Pesquisa em Saúde (national registration number CAAE 20225713.5.0000.5320). Consent forms and questionnaires were kept separately in a laboratory located in a State other than that where patients lived. The names of the patients could not be linked to any study data collected.

### Subjects

Blood samples were collected in 2013 from 202 adults attending the viral hepatitis ambulatory of the Department of Health of Chapecó County, State of Santa Catarina, southern Brazil. All of them were HBsAg positive patients, diagnosed as HBV chronic carriers between 1991 and 2013. Four (2%) and two (1%) of them were anti-HIV and HCV RNA positive, respectively. Information about HBV serological status and treatment of the patients, as well as date of notification of the disease, was collected in medical records. Additionally, 161 HBsAg negative, anti-hepatitis B core (anti-HBc) positive individuals were recruited among (i) those who had been taken care in the past in the ambulatory, (ii) family members of the above-described chronic carriers, and (iii) health care workers of community health centers of Chapecó County.

### Viral DNA extraction

Viral DNA extraction was performed from 200 μL of plasma by using the HiYield Viral Nucleic Acid Extraction kit (RBC Bioscience, Taipei, Taiwan) according to the manufacturer's instructions, with the following modifications: 0.5 mg/mL of proteinase K was added to the lysis buffer, and lysis was performed for 15 min at 60°C. DNA was recovered in 50 μL of DNase/RNase-free water and stored at—20°C.

### Calculation of viral load, nucleotide sequencing, subtyping and genotyping

HBV DNA detection and quantification was performed by TaqMan real-time polymerase chain reaction (PCR). After alignment of HBV sequences available in the GenBank database by using Clustal X (Conway Institute, Dublin, Ireland), BioEdit (Abbott Company, Carlsbad, CA) and PrimerExpress (Applied Biosystems, Foster City, CA) softwares, a consensus sequence was obtained which was used to design forward (5’-TTGTCCTGGYTATCGYTGGATGTG-3’) and reverse (5’-GATGAGGCATAGCAGCAGGATG-3’) PCR primers and fluorescent probe (6-FAM-TGCGGCGTTTTATCAT-MGB-NFQ). The PCR product was a 72-base-pair (bp) fragment of the surface antigen gene. PCR assay was performed on an ABI 7500 platform (Applied Biosystems) in a 30 μL reaction containing 9 μL of DNA template, TaqMan universal master mix (Applied Biosystems), 300 nM of each primer and 250 nM of probe. Samples with known viral load were used as controls. Samples and controls were tested in triplicate. The test was linear from 1.0 log IU/mL to 8.0 log IU/mL, and the reaction efficiency was 98.3%.

HBV DNAs of the real-time PCR positive samples were then amplified by using a conventional PCR assay described previously [17], which generated a 485-bp fragment located in the S region of the genome. Amplicons were purified with PureLink PCR Purification Kit (Life Technologies, Carlsbad, CA). Nucleotide sequencing was done in both directions with BigDye Terminator v3.1 Cycle Sequencing Kit, and sequencing reactions were analyzed on a 3130xl Genetic Analyzer (Applied Biosystems).

Deduced amino acid sequences were used to predict the subtypes of the HBV isolates by determination of the residues present at positions 122, 127, 134 and 160 of the S protein, as described previously [18].

Genotypes of the HBV isolates were first determined by using the BLAST algorithm (http://blast.ncbi.nlm.nih.gov/Blast.cgi) which calculates the percent identities between a given query nucleic acid sequence and a database of sequences of known genotypes. Moreover, the high-quality portion (358 bp) of the sequences were aligned with those of 231 genotype D isolates, i.e. all those which were completely sequenced (3,182 bp) and whose subgenotype was informed in Genbank. Reference strains belonging to the other genotypes were included in the alignment. This was performed by using Clustal-X from MEGA software version 6 [19]. Maximum likelihood method was used to construct a phylogenetic tree.

### Genealogical origins of the patients

All but one patient were born in Brazil, and the one remaining was Haitian. However, it was noticed that many patients had not Portuguese-sounding surnames, as it is usual in Brazil, due to Portuguese colonization. It was therefore decided to conduct a genealogical research using the surnames of the HBV infected patients to determine their ancestry. This was conducted in four, freely accessible dababases containing a very large number of family names, in order to determine the geographical origin of the surnames of the patients. In all four databases, the search queries were the own family names of the patients. Only exact matches were considered, excluding close matches and alternative spellings. However, the object of the search varied from one database to another. Database *MyHeritage* (www.myheritage.com), whose purpose is to reconstruct the history of the families, was queried for the most common among the birth countries of the persons carrying the same last names as those of patients. As most non-indigenous Brazilians are descendant of Europeans, only European countries were considered. Similarly, the database of the *Statue of Liberty-Ellis Island Foundation* (www.libertyellisfoundation.org), which identifies passengers of the ships that brought immigrants to The United States, was screened for the most common last residence/birth country of the people carrying the surnames of the patients. The presence of those family names was also investigated in the database of the *Ferrara Cidadania Italiana* company (www.ferraracidadaniaitaliana.com.br), which includes names of Italians who immigrated in Brazil. Finally the *Cognomix* database (www.cognomix.it), which shows the geographical distribution of surnames within Italy, was used to determine the regions of Italy where each surname is more disseminated.

In addition, all patients were asked about the places of birth of their parents and grandparents as well as the countries of origin of the families of their four grandparents.

### Statistical analysis

Categorical variables of epidemiological features were compared using Pearson’s χ^2^ test or Fisher’s exact test, as appropriate. Continuous variables were compared using ANOVA. *P values* < 0.05 were considered statistically significant. Data were analysed using SPSS 20.0 software (IBM, Armonk, NY).

## Results

### Characteristics of the patients

Table 1 shows the demographic, epidemiological and serological characteristics of 202 HBV chronically infected patients attending in 2013 the viral hepatitis ambulatory of Chapecó, southern Brazil. Data were compared with those of similar, although smaller, groups of patients of the same ambulatory notified in 1996 and 2006, respectively, whose data have been published previously [20]. The male:female ratio was almost identical (54.5–56.0% of males) in the three groups. An aging of the chronically HBV infected population over the years was observed: while about two-thirds of the patients were 20–39 years old in 1996 and 2006, more than 60% were aged 40 to 59 years in 2013. Of note, the mean age at notification (newcomers) increased significanly, from 29.9 years in 1996 to 34.9 in 2006 and 44.4 in 2011–2013 (p < 0.01). Patients were primarily (87.6%) whites, without significant variation of the proportion over time. Vertical transmission was the major risk factor identified, with 24.8% of patients whose mother and/or siblings were HBV carriers, followed by sexual and parenteral transmission. Hepatitis B ‘e’ antigen (HBeAg) marker was detected in a small proportion (8.4%) of the patients, as it was the case for the groups studied in 1996 and 2006 (Table 1).

**Table 1:** Demographic, epidemiological and serological characteristics of HBV chronically infected patients accompanied in Chapecó, southern Brazil (1996, 2006 and 2013).

Feature	2013 (this work)	2006 [20]	1996 [20]	*p* value
				
Number of patients	202	66	84	
Males	110 (54.5%)	36 (54.5%)	47 (56.0%)	NS
Age range, years				<0.01
*18–19*	1 (0.5%)	4 (6.1%)	13 (15.5%)	
*20–39*	55 (27.2%)	42 (63.6%)	59 (70.2%)	
*40–59*	124 (61.4%)	18 (27.3%)	11 (13.1%)	
*≥ 60*	22 (10.9%)	2 (3.0%)	1 (1.2%)	
Age at notification (newcomers only)	44.4 ± 13.3 ^a^	34.9 ± 11.9	29.9 ± 10.3	<0.01
Ethnicity				NS
*Whites*	177 (87.6%)	63 (95.5%)	77 (91.7%)	
*Blacks or mulattos*	25 (12.4%)	3 (4.5%)	6 (7.1%)	
Risk factor				-
*Vertical*	50 (24.8%)	n.a.	n.a.	
*Parenteral*	36 (17.8%)	n.a.	n.a.	
*Sexual*	22 (10.9%)	n.a.	n.a.	
*Unknown*	94 (46.5%)	-	-	
Serological markers				
*HBeAg*	17 (8.4%)	8 (12.1%)	2 (2.4%)	NS
*Anti-HBe*	170 (84.1%)	n.a	n.a	
*Anti-HBs*	0	n.a.	n.a.	

NS, not significant; n.a. not available

^a^ Based on the 64 patients notified between 2011 and 2013.

### Viral load and occult infection

DNAs extracted from all 363 samples of this study, including 161 anti-HBc positive, HBsAg negative samples, were submitted to real time PCR. On a total of 202 HBsAg positive samples, 120 (59.4%) gave positive results, with a proportion slightly higher in the group of untreated patients (63.2%) than among those under treatment (51.5%) (Table 2). No significant difference of mean viral load was noted between the two groups (3.0 vs. 3.1 log IU/mL). As expected, a strong positive correlation (*p* = 0.0015) was observed between detection of HBV DNA and HBe antigenaemia (not shown). Interestingly, nine out of the 161 anti-HBc positive, HBsAg negative samples gave PCR positive results, that suggested the occurrence of occult infection, although at low (5.6%) frequency and viral load (mean 2.1 log IU/mL).

**Table 2:** HBV DNA detection and viral load in HBsAg positive and negative subjects.

	HBsAg positive		
HBV DNA detection	Under treatment (n = 66)	Not treated (n = 136)	Anti-HBc positive, HBsAg negative (n = 161)
Real-time PCR positive samples	34 (51.5%)	86 (63.2%)	9 (5.6%)
Viral load of positive samples			
*< 100 IU/mL*	11	14	2
*100–2000 IU/mL*	13	51	7
*> 2000 IU/mL*	10	21	0
*Mean ± SD (log IU/mL)*	3.1 ± 1.7	3.0 ± 1.3	2.1 ± 0.3

### Genotypes and subtypes distribution

Direct sequencing of the S region, and subsequent subtyping and genotyping, were achieved for 91 HBsAg positive samples. Table 3 summarizes the results. Three (3%) and 88 (97%) samples belonged to genotypes A and D, respectively. Within genotype A, the two isolates derived from patients born in Brazil were classified as *adw2*, while the third one, from the only patient born out of Brazil (Haiti), was *ayw1*. Within genotype D, 60, 21 and 5 HBV isolates were subtyped as *ayw2*, *ayw3* and *ayw4*, respectively. Two additional *ayw* samples could not be fully subtyped, due to the presence of an Ala residue at position 127 of the small S protein in place of the usual residues Pro for subdeterminant *w1/2*, Thr for *w3*, or Leu for *w4*. Moreover, one of these two *ayw* samples showed base ambiguities at several genome positions, suggesting a mixed infection with two HBV/D isolates.

**Table 3:** Distribution of HBV genotypes and serological subtypes among HBV chronically infected patients.

Genotype	Subtype	*n*	Birth country
A	All	3	
	*adw2*	2	Brazil
	*ayw1*	1	Haiti
D	All	88	
	*ayw2*	60	Brazil
	*ayw3*	21	Brazil
	*ayw4*	5	Brazil
	*ayw* ^*a*^	2	Brazil
Total		91	

^a^ not fully subtyped due to the presence of an alanine residue at position 127 of the small S protein

**Fig 1:**
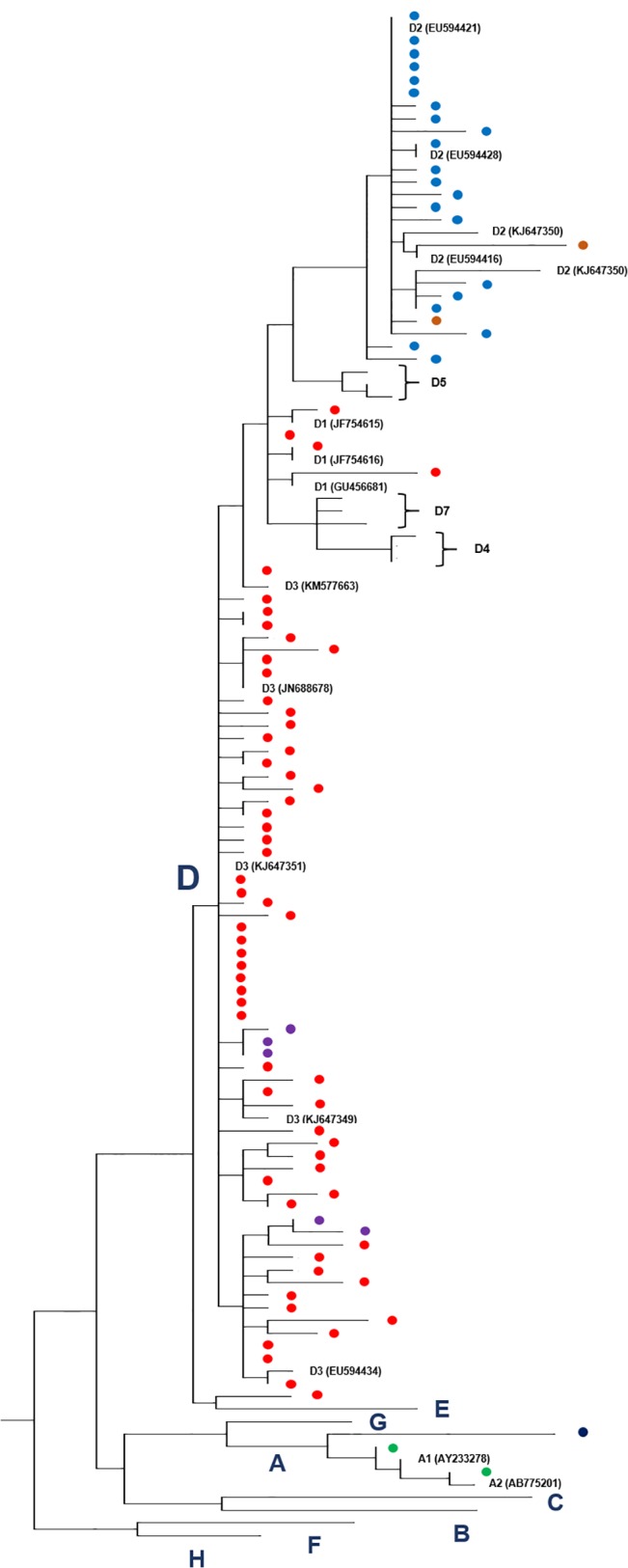
Phylogenetic analysis based on HBV small S nucleotide sequences. The phylogenetic tree, performed by using the maximum likelihood method, incorporates 30 isolates which sequences are available in GenBank along with the 91 isolates from this study represented by filled circles. The following color-code indicates serotypes: green, *adw2*; dark blue, *ayw1*; red, *ayw2*; light blue, *ayw3*; purple, *ayw4*; brown, *ayw*. The GenBank accession numbers not indicated on the figure are: Genotype B, D00329; C, AB112066; D4, KF192838, KF192840 and KF192841; D5, GQ205378, GQ205379 and GQ205389; D7, FJ904430, FJ904444 and FJ904447; E, X75664; F, X69798; G, AB056513; H, AY090454.

A phylogenetic tree is shown in Fig 1, that was constructed with the 91 sequences determined in this study along with 30 sequences available in GenBank and representative of the different HBV genotypes and HBV/D subgenotypes. Although it was judged preferable to restrict the classification of the isolates from Chapecó to the genotype-, not subgenotype level, because partial sequencing of the HBV genome may not be appropriate to ascertain the subgenotype [21,22], it could be observed that a majority (60/91) of them were closely related to isolates characterized as D3 after sequencing of their complete genomes. A good correlation could be observed between genotype D2 and subtype *ayw3* (blue filled circles) on one hand, and between genotype D3 and subtype ayw2 (red) on the other hand.

### Origin of the patient family names

Genotypes HBV/A and HBV/F have been described as predominant in Brazil [13,23] and the other South American countries [14], respectively. While seeking the reason why genotypes A and F were absent or almost absent in the group under study (97% of HBV/D), it was noticed that many patients had not Portuguese-sounding family names, as it is generally the case in Brazil, due to Portuguese colonization. In an attempt to unveil the origin of HBV/D, it was then decided to search for the geographical origins of the surnames of all the 88 HBV/D infected patients. This search was performed in four online, freely accessible databases, each of them created with a different purpose (Table 4). By retrieving data from the two largest databases, *MyHeritage* (family history; 1.7 x 10^9^ profiles) and *Statue of Liberty-Ellis Island Foundation* (immigrants in the USA; 5.1 x 10^7^ ship passenger records), Italy was found to be the most common country of birth (or last residence) of the persons carrying the surnames of the patients (43/88 [49%] and 36/73 [49%] cases, respectively). Moreover, the surnames of 34 (39%) patients were found among the records of Italian immigrants in Brazil, compiled in the *Ferrara Cidadania Italiana* database. From the *Cognomix* database, it was infered that Veneto was the region of Italy where the surnames of the patients were the most disseminated, in agreement with historical records of Italian immigration in Brazil [24].

**Table 4:** Geographical origins of the surnames of 88 HBV/D infected patients living in Chapecó, southern Brazil, traced from four free databases.

Database						Results	
Name	Countries	Purpose (search for)	Number of records	Research tool	No. (%) of patients surnames present in the database	Searched item	Countries/ regions of Italy
MyHeritage	All	Family history	1.7 x 10^9^ profiles	‘Supersearch’	88 (100%)	Most common birth country^a^	Italy, 43 Portugal, 11 Others, 34
Statue of Liberty-Ellis Island Foundation	All	Immigrants in the USA	5.1 x 10^7^ ship passengers	‘Passenger search’	73 (83%)	Most common last residence/ birth country^a^	Italy, 36 Portugal, 18 Others, 19
Ferrara Cidadania Italiana	Italy	Italian immigrants in Brazil	3.5 x 10^5^ records	‘Search your surname’	34 (39%)	–	–
Cognomix	Italy	Italian surnames	1.1 x 10^4^ surnames	‘Maps of Italian surnames’	48 (54%)	Geographical distribution of the surnames in Italy^b^	Veneto, 17 Lombardy, 6 Others, 5

In all searches, only exact matches were considered, excluding close matches and alternate spellings.

^a^ Only European countries were considered.

^b^ Only surnames carried by more than one hundred people were considered.

### Genealogical origins of the patients

A survey was then conducted directly with the 88 HBV/D infected patients who were asked about the places of birth of their parents and grandparents as well as the countries of origin of the families of their four grandparents. These questions were answered by 87 (all but one) patients. However, four of them did not know the origin of any of their grandparents. Table 5 shows the results obtained from the remaining 83 patients. Interestingly, 43 (52%) patients declared that their four grandparents were of Italian origin, vs. only seven (8%) who had their four grandparents originated from Brazil. At all, 65 out of 83 (78%) patients had at least one grandparent originating from Italy, compared with 29 (35%), 11 (13%), six (7%) and two (2%) from Brazil, Germany, Portugal and Poland, respectively. Finally, three (4%) patients declared that one of their grandparents was indigenous.

**Table 5:** Countries of origin of the families of the grandparents of HBV/D infected patients living in Chapecó, southern Brazil.

Origins of the families	*n*
**Italy (4)**	**43**
**Italy (3), Brazil (1)**	**2**
**Italy (3), Germany (1)**	**1**
**Italy (3), Poland (1)**	**1**
**Italy (2), Brazil (2)**	**3**
**Italy (2), Portugal (2)**	**1**
**Italy (2), unknown (2)**	**2**
**Italy (2), Germany (1), indigenous (1)**	**1**
**Italy (2), Brazil (1), unknown (1)**	**1**
**Italy (1), Brazil (3)**	**1**
**Italy (1), Portugal (3)**	**2**
**Italy (1), Brazil (2), Germany (1)**	**1**
**Italy (1), Brazil (2), unknown (1)**	**1**
**Italy (1), Brazil (1), unknown (2)**	**1**
**Italy (1), Brazil (1), Germany (1), Portugal (1)**	**1**
**Italy (1), Brazil (1), Germany (1), unknown (1)**	**1**
**Italy (1), unknown (3)**	**2**
**Brazil (4)**	**7**
**Brazil (3), Portugal (1)**	**1**
**Brazil (3), unknown (1)**	**1**
**Brazil (2), Germany (2)**	**1**
**Brazil (2), unknown (2)**	**2**
**Brazil (2), Germany (1), Portugal (1)**	**1**
**Brazil (2), Germany (1), indigenous (1)**	**1**
**Brazil (2), Germany (1), unknown (1)**	**1**
**Brazil (1), Poland (3)**	**1**
**Brazil (1), Germany (1), indigenous (1), unknown (1)**	**1**
**Germany (4)**	**1**
**Total**	**83**

Patients were asked about the countries of origin of their four grandparents families. All four may originate from the same country or not. Brazil, Brazilian non-indigenous families.

## Discussion

The city of Chapecó, founded in 1917, is located in the western part of the State of Santa Catarina, a region of southern Brazil colonized by Italian, German and other European immigrants. In Chapecó, the prevalence of HBsAg and anti-HBc has been reported to be 2- to 10-fold higher than in other cities of the State [4]. In this study, demographic data of HBV chronic carriers, accompanied in 2013 at the hepatitis ambulatory of Chapecó, were compared with those obtained in the same ambulatory in 1996 and 2006 [20]. While neither the male:female ratio nor the distribution by ethnic groups varied significantly, a very fast increase was observed with respect to the mean age at which patients were directed to the ambulatory and notified of their disease, from 29.9 years in 1996, 34.9 in 2006 and 44.4 in 2011–2013. This rapid increase likely resulted from the hepatitis B compulsory vaccination campaign initiated in 1994 in Chapecó, which targeted not only babies but also children and adolescents of school age [25], thus reducing considerably the proportion of HBV infected young adults (< 35 years old) in the last two decades.

A low percentage (15.3%) of HBV carriers, whether under treatment or not, showed a viral load > 2000 IU/mL, consistent with the low proportion (8.4%) of HBeAg positive patients. Otherwise, 9/161 (5.6%) anti-HBc positive, HBsAg negative subjects (serological pattern typical of past infection) tested positive for HBV DNA by real time PCR, suggesting the occurrence of occult B infection [26]. The HBV DNA positivity rate in this group was higher than that (3.3%) found among candidate blood donors from the same geographic region whose blood was rejected due to anti-HBc reactivity [17], although the difference was not statistically significant (*p* > 0.05).

HBV isolates have been classified in at least eight genotypes (A–H) and nine main serological subtypes (*adw2*, *adw4*, *ayw1*, *ayw2*, *ayw3*, *ayw4*, *ayr*, *adrq* ^*+*^ *and adrq* ^*-*^ *)* [9,18]. HBV genotype A has been shown to be more sensitive to interferon treatment than HBV/D [27,28], and interferon has been suggested as first-line therapy in all genotype A patients [12]. Genotyping of HBV isolates may thus be a valuable tool to support therapeutic decisions, particularly in countries or regions where both genotypes A and D circulate, and where interferons are considered as a treatment option, as it is the case in Brazil. On a total of 91 HBV isolates genotyped in this study, 88 (97%) were from genotype D. Such a high percentage of genotype D is unusual in South America, where HBV/A (in Brazil) and HBV/F (in the other countries) are the most prevalent [13,14,23]. This peculiar condition should constitute a strong evidence to guide the treatment decisions to be taken by the health authorities for patients of Chapecó and surrounding region.

Genotype D includes mainly *ayw2* and *ayw3* isolates, which are predominant in Italy and other Mediterranean countries. Here, 81/88 HBV/D isolates were predicted to be *ayw2* and *ayw3*, likely related to isolates circulating in Italy (see below). The *‘w’* sub-determinant could not be defined for two *ayw* isolates. Both showed the atypical substitution Ala127 in the small S protein, previously found in a Russian isolate [29]. Moreover, one of them was associated with another HBV/D isolate (mixed infection). The remaining five HBV/D isolates were *ayw4*. Worldwide, most *ayw4* isolates belong to genotype E. To our knowledge, the D/ayw4 isolates characterized in this study are the first reported so far in South America. However, D/*ayw4* isolates have already been described in North Africa [30] and the Middle East [31]. In Brazil, about eleven million people (5–6% of population) are descendents from Lebanese, Syrian and other Arab immigrants who arrived in the 19^th^ and 20^th^ centuries. Whether D/*ayw4* isolates were introduced in Brazil through Arab immigration deserves further investigation.

HBV genetic variability has been useful in epidemiological and transmission studies, tracing human migrations [9,15]. In South America, where genotypes A and F predominate, the three states of southern Brazil, namely Paraná, Rio Grande do Sul and Santa Catarina, seem to be an exception. Indeed, high (67–100%) proportions of genotype D have been reported in different cities of these states [14,32–35]. A recent report has proposed that HBV/A1 strains, predominant in Brazil, have been brought by the slaves removed from Southeast Africa at the middle of the 19th century [15]. Complementarily, the present study intended to investigate how HBV/D was introduced in southern Brazil. In the late nineteenth and early twentieth centuries, large numbers of European immigrants arrived in southern Brazil. The existence of ‘islands’ of enhanced HBsAg prevalence (1.5–3% vs. 0.5% countrywide), such as Chapecó [4]. and Caxias do Sul [5], in a region recently colonized by European immigrants, as well as the fact that genotype D is one of the most prevalent in Europe [36–39], may suggest that HBV/D was imported into South America through that immigration. At this respect, Bertolini and collaborators [40] have mentioned an elevated HBV prevalence among Brazilian women of Italian and German descent, and suggested that the high prevalence of HBV/D in the south of Brazil was due to the intense migration of settlers from European countries [16]. However, this hypothesis has not been well documented so far.

In this study, a search for the geographical origins of the surnames of 88 HBV/D infected patients living in Chapecó, southern Brazil, was performed in an attempt to unveil the origin of genotype D in Brazil. The use of online family names databases to search for the geographic origin of viruses is a novelty. In practice, these tools do require neither a large amount of personal information (only patient family names are necessary) nor purchasing of any package, since access is free. Taken into consideration the lack of consensus on the HBV evolutionary rate that makes it difficult to reconstruct the timescale of the HBV origin [11], the method used in this study of searching for the geographical origins of the virus through human migrations may constitute a valuable complement to phylogeny and phylogeography studies.

Data obtained by screening of two large databases (*MyHeritage* and *Statue of Liberty-Ellis Island Foundation*) showed that Italy was by far the most common country of birth (or last residence) of the persons having the same surnames as the patients (approximately 50% of the cases) (Table 4). Moreover, the surnames of 34 (39%) patients were found among the records of Italian immigrants in Brazil in the *Ferrara Cidadania Italiana* company database. Noteworthy, this number was probably underestimated because (i) only 350,000 of the 1.4 million Italian people who immigrated in Brazil between 1870 and 1920 [41,42] have been registered in that database and (ii) many surname transcription mistakes were made both at the entrance of immigrants and at the time of birth registration of their descendants. Veneto and Lombardy, in this order, were the regions of Italy where the surnames of the patients appeared to be most disseminated, consistent with historical records of Italian immigration in southern Brazil [24]. Furthermore, when asked about the geographical origins of their grandparents, 52% of the HBV/D infected patients answered that their four grandparents had an Italian origin, and 78% had at least one grandparent originating from Italy.

HBV subgenotype D3 has been reported as the most prevalent in Italy [38,43], one of the few countries where this happens. Although partial sequencing of the viral genome may not be appropriate to ascertain HBV subgenotypes, it is interesting to note that the majority (60/91) of HBV isolates from Chapecó were closely related to D3 isolates. Thus, the data set collected in this study strongly suggested that a large proportion of the HBV/D isolates circulating in southern Brazil were introduced through the Italian immigration, which culminated between 1870 and 1920. However, as there have been a lot exchanges between Brazil and Europe, it cannot be excluded that some HBV/D isolates were introduced from other countries.

The prevalence and distribution of the different HBV genotypes in Brazil are largely the result of settlement. The fact that genotype F is widespread in the indigenous populations of South America attests to its presence in pre-Columbian times. Logically, the other genotypes should have been brought by the successive waves of colonization (or deportation in the case of the slaves). So, HBV/A1 would have been introduced from Southeastern Africa by illegal slave trafficking in the mid-nineteenth century [15], and HBV/D entered in southern Brazil through Italian immigration in the late nineteenth and early twentieth centuries (this study). Assuming that (i) the prevalence of HBsAg carriers was around 3% in the Italian general population in the pre-vaccination era [44], and (ii) half of the 1.4 million Italians who emigrated to Brazil between 1870 and 1920 settled in the three states of southern Brazil, the number of HBV infected Italian people who arrived in Southern Brazil can be estimated to 21,000, i.e. 1.5–2% of the local population at the time. The fact that the immigrant population concentrated in some counties could explain the persistence, until now, of ‘islands’ of elevated prevalence.

Three D3 sequences (accession numbers JN688678, KJ647351 and KJ647349), closely related to Brazilian sequences (Fig 1), were from Argentinian isolates. Italians were by far the most numerous immigrants in Argentina during the 19^th^ and 20^th^ centuries. It is therefore possible that a number of HBV/D isolates circulating in Argentina are also of Italian origin.

Barros and collaborators [45] recently reported proportions of 67% and 28% of genotypes A1 and D, respectively, among chronically HBV infected patients living in the State of Maranhão, Northeastern Brazil. Among HBV/D isolates, subgenotype D4 was predominant. The authors suggested that this subgenotype has been introduced in Maranhão by means of the slave trade during the late 18^th^ century. Further studies are needed to confirm the assumption that the HBV/D isolates circulating in Brazil came from two different continents (Europe and Africa).
